# Oral *P. gingivalis* impairs gut permeability and mediates immune responses associated with neurodegeneration in LRRK2 R1441G mice

**DOI:** 10.1186/s12974-020-02027-5

**Published:** 2020-11-19

**Authors:** Yu-Kun Feng, Qiong-Li Wu, Yan-Wen Peng, Feng-Yin Liang, Hua-Jing You, Yi-Wei Feng, Ge Li, Xue-Jiao Li, Shu-Hua Liu, Yong-Chao Li, Yu Zhang, Zhong Pei

**Affiliations:** 1 Department of Neurology, The First Affiliated Hospital, Sun Yat-sen University; Guangdong Provincial Key Laboratory of Diagnosis and Treatment of Major Neurological Diseases, National Key Clinical Department and Key Discipline of Neurology, No.58 Zhongshan Road 2, Guangzhou, 510080 China; 2 Department of Neurology, Hainan General Hospital; Hainan Affiliated Hospital of Hainan Medical University, Haikou, 570311 Hainan China; 3 Department of Immunology, Zhongshan School of Medicine, Sun Yat-sen University, Guangzhou, 510080 China; 4 The Biotherapy Center, the Third Affiliated Hospital, Sun Yat-sen University, Guangzhou, 510630 China; 5 Department of Neurology, Huashan Hospital, Fudan University, Shanghai, 200000 China; 6 Guangdong Laboratory Animals Monitoring Institute, Guangdong Provincial Key Laboratory of Laboratory Animals, Guangzhou, 510663 Guangdong China

**Keywords:** Chronic periodontitis, Parkinson’s disease, Dopaminergic neurons, R1441G LRRK2, IL-17A

## Abstract

**Background:**

The R1441G mutation in the leucine-rich repeat kinase 2 (LRRK2) gene results in late-onset Parkinson’s disease (PD). Peripheral inflammation and gut microbiota are closely associated with the pathogenesis of PD. Chronic periodontitis is a common type of peripheral inflammation, which is associated with PD. *Porphyromonas gingivalis* (Pg), the most common bacterium causing chronic periodontitis, can cause alteration of gut microbiota. It is not known whether Pg-induced dysbiosis plays a role in the pathophysiology of PD.

**Methods:**

In this study, live Pg were orally administrated to animals, three times a week for 1 month. Pg-derived lipopolysaccharide (LPS) was used to stimulate mononuclear cells in vitro. The effects of oral Pg administration on the gut and brain were evaluated through behaviors, morphology, and cytokine expression.

**Results:**

Dopaminergic neurons in the substantia nigra were reduced, and activated microglial cells were increased in R1441G mice given oral Pg. In addition, an increase in mRNA expression of tumor necrosis factor (TNF-α) and interleukin-1β (IL-1β) as well as protein level of α-synuclein together with a decrease in zonula occludens-1 (Zo-1) was detected in the colon in Pg-treated R1441G mice. Furthermore, serum interleukin-17A (IL-17A) and brain IL-17 receptor A (IL-17RA) were increased in Pg-treated R1441G mice.

**Conclusions:**

These findings suggest that oral Pg-induced inflammation may play an important role in the pathophysiology of LRRK2-associated PD.

**Supplementary Information:**

The online version contains supplementary material available at 10.1186/s12974-020-02027-5.

## Background

Parkinson’s disease (PD) is the second most common neurodegenerative disease that results in a progressive movement disorder characterized by slowness, rigidity, gait difficulty, and rest tremors [1]. Degeneration of dopaminergic neurons in the substantia nigra pars compacta (SNpc) is one of the pathological hallmarks of PD [2, 3]. Although the exact cause of PD remains poorly understood, it is generally believed that complex interactions between genetic and environmental factors contribute its development.

Leucine-rich repeat kinase 2 (LRRK2) mutants are the most common genetic factors in the pathogenesis of PD [4]. Substantial evidence suggests that mutant LRRK2 strongly activates brain immune cells, which in turn mediate neurodegeneration through neuroinflammation [5, 6]. Interestingly, LRRK2 has been also linked to several systemic inflammatory diseases, such as inflammatory bowel disease and leprosy [7, 8]. Activation of LRRK2, however, has been reported to induce opposite effects in the brain and the periphery. For example, activation of LRRK2 protects against infection in the gut, but causes neurodegeneration in the brain [9, 10].

Recently, chronic systemic inflammatory diseases have been linked to the risk of developing PD. Periodontal disease is a common chronic inflammatory disease and is associated with PD [11–13]. Interestingly, *Porphyromonas gingivalis* (Pg), the major periodontal pathogen, induces dysbiosis of gut microbiota [14, 15]. The relationship between intestinal function disorder and PD has attracted much attention [16, 17]. Until now, the link between the two diseases was based on motor disturbances caused by PD, which could lead to progression of periodontal disease [12, 13]. Recently, the major virulence factors of *P. gingivalis* such as gingipain R1 and lipopolysaccharide (LPS) have been detected in the blood stream in PD patients [18, 19]. However, whether periodontal disease can have an influence on initiation and progression of PD through the intestinal pathway and the underlying mechanism remains unclear.

Recent studies suggest that peripheral lymphocytes may play a central role in the pathophysiology of PD [20]. For example, interleukin-17A (IL-17A) level was significantly increased in the serum of patients with PD [20–22]. Furthermore, IL-17A could induce human induced pluripotent stem cell-derived midbrain neuronal cell death, possibly through IL-17 receptor A (IL-17RA) [20, 21]. IL-17A is mainly driven by Th17 lymphocytes. Interestingly, Th17 cells have been linked to several immune-related diseases, including periodontal disease [23, 24]. Th17 cells are also essential for normal defense against gut pathogens [25].

Therefore, we hypothesized that oral Pg might induce peripheral inflammatory responses leading to degeneration of dopaminergic neurons through the gut in LRRK2 R1441G mice.

## Materials and methods

### Animals

All animal procedures were performed according to the Guide for the Care and Use of Laboratory Animals of Sun Yat-sen University (Guangzhou, China). All animals were housed in a specific pathogen-free facility with a 12:12-h light/dark cycle, ad libitum food and water. In this study, 3- to 4-month-old FVB/NJ and FVB/N-Tg (LRRK2*R1441G)135Cjli/J mice were purchased from the Jackson Laboratory (Bar Harbor, ME, USA) and crossed in the Guangdong Laboratory Animals Monitoring Institute (Guangzhou, China). At 1 month, all littermates were genotyped. Genotyping was done by polymerase chain reaction (PCR) of tail DNA using a protocol from the Jackson Laboratory. A total of 40 mice were used in this study and assigned to four groups: FVB/N + carboxymethyl cellulose (F + C), FVB/N + Pg (F + Pg), R1441G + C, and R1441G + Pg.

### Pg cultures and administration

Pg was cultured in broth (Brain Heart Infusion, l-cysteine hydrochloride monohydrate, yeast extract, and chloroproto-ferriheme, Sigma-Aldrich, St. Louis, MO, USA). After that, Pg was placed in an anaerobic container for 48 h at 37 °C. A total of 10^9^ colony-forming units of live Pg was suspended in 0.1 ml phosphate-buffered saline (PBS) with 2% carboxymethyl cellulose (CMC) (Sigma-Aldrich) and given to each mouse by gavage three times a week for about a month, as described previously [14, 15]. The control group was administered 0.1 ml PBS with 2% CMC without Pg. After administration, all mice were allowed to eat and drink ad libitum.

### Behavioral tests

#### Rotarod test

Animals were placed on an accelerating rotarod (Xin Ruan, Shanghai, China) with an accelerated speed of 4–40 rpm for 5 min, and the latency to fall was recorded each time. Animals were tested three times a day for three consecutive days, allowing for 2 days of training and acclimatization. A resting time of at least 30 min was given between trials. The results are presented as the average of the three times.

#### Open field

Animals were placed in the chamber (45 × 45 × 45 cm) with a video camera (Xin Ruan, Shanghai, China). Every mouse was carefully placed in the center of the chamber and allowed to freely explore the chamber. Animals were tested for two consecutive days, allowing for 1 day of training and acclimatization. The movement of mice was filmed and analyzed automatically for 10 min.

### Immunofluorescence

The brain tissue and colon were removed, fixed, and dehydrated to further process for immunofluorescence. After blocking for 1 h at room temperature, brain sections were incubated with primary antibodies overnight at 4 °C. The primary antibodies used in this study were tyrosine hydroxylase (TH) (MAB318, Millipore, Bedford, MA, USA), allograft inflammatory factor 1 (Iba1) IgG (019-19741, Wako, Japan), cleaved active caspase-3 (9661, Cell Signaling Technology, Danvers, MA, USA), LRRK2 (MJFF2 [c41-2]) (ab133474, Abcam, Cambridge, UK), MAP2 lgG (ab32454, Abcam), Iba1 lgG (MA5-27726, Thermo Fisher Scientific, Waltham, MA, USA), and IL-17RA lgG (ab180904, Abcam). Subsequently, the sections were incubated with Alexa 488- or 555-conjugated secondary antibodies (4408, 4413, Cell Signaling Technology) for another 1 h at room temperature. Finally, sections were viewed under a Nikon microscope (Japan). The number and density of cells were measured in a double-bind manner with ImageJ v1.51 software and two individuals. For TH+ cell counting, five consecutive sections containing the substantia nigra were selected per animals.

### Western blot

The brain was cut into sections in the mold. The colon and the SN tissue were homogenized in radioimmunoprecipitation assay buffer (Thermo Scientific) with phenylmethanesulfonyl fluoride (1:100) and phosphatase inhibitors (Roche, Basel, Switzerland) in an ultrasonic disintegrator. Homogenates were incubated on ice for 30 min and centrifuged at 12,000 rpm for 25 min at 4 °C. The protein concentration was determined using the Pierce BCA Protein Assay Kit (Thermo Scientific). Equal quantities of proteins were separated by sodium dodecyl sulfate polyacrylamide gel electrophoresis and transferred to polyvinylidene fluoride membranes. The membranes were blocked with 5% non-fat dry milk or bovine serum albumin for 1 h at room temperature and incubated with primary antibody overnight at 4 °C. The primary antibodies used were LRRK2 (MJFF2 [c41-2]) lgG (ab133474, Abcam), phospho-LRRK2^S935^ lgG (ab133450, Abcam), MAP2 lgG (ab32454, Abcam), cleaved active caspase-3 lgG (BF0711, Affinity, China), and IL-17RA lgG (ab180904, Abcam). α-Tubulin was used as a loading control. After incubating with anti-rabbit or anti-mouse secondary antibodies (7074, 7076, Cell Signaling Technology) for 1 h, the bands were visualized using the electrochemiluminescence detection reagents (Millipore) on an Amersham Imager 600 (Amersham Biosciences, USA). The relative density of protein was analyzed by ImageJ v1.51 software.

### Mononuclear cell cultures and stimulation

The spleens were mechanically disrupted and filtered through a 40-μm cell-strainer (Falcon, BD Biosciences, Durham, NC, USA) and isolated by Ficoll-Hypaque (Tianjin HaoYang Biological Manufacture Co, Ltd, Tianjin, China) density gradient centrifugation to procure mononuclear cells, according to the manufacturer’s instructions. The cells (2 × 10^6^/ml) were suspended in complete Roswell Park Memorial Institute 1640 medium and stimulated for 24 h with or without Pg-LPS (1 μg/ml; SMB00610, Sigma-Aldrich) in the presence of anti-CD3 mAb and anti-CD28 mAb (553057, 553294, BD Biosciences) in round-bottomed 96-well plates (200 μl/well) at 37 °C and 5% CO_2_.

### Enzyme-linked immunosorbent assay (ELISA)

The levels of IL-17A in the supernatant from Pg-LPS-stimulated mononuclear cells were measured with an ELISA kit (88-7371, BioLegend, CA, USA). ELISA assays were performed according to the manufacturer’s instructions. Data were collected by an ELISA reader under a wavelength of 450 nm. The results are shown as the mean readings from triplicate wells.

### Multiplex cytokine and chemokine analysis

Peripheral blood was acquired through retro-orbital bleeding. The whole blood was placed at room temperature for 1 h and centrifuged at 1000*g* for 20 min. The supernatant was taken to obtain the serum. Serum samples, reagents, and standards were prepared according the instruction of the Multi-Analyte Flow Assay Kit (BioLegend, CA, USA). Briefly, standards and all diluted samples were added into 96 V-bottom Plate wells in the presence of mixed beads and then shook at 800 rpm for 2 h at room temperature. Twenty-five-microliter detection antibodies were added into each well and incubated about 1 h at room temperature. Then, 25-μl SA-PE were added to each well. The beads were resuspended and analyzed on a flow cytometer (Becton Dickinson, San Jose, USA). The data was analyzed by LEGENDplex software (TreeStar, San Carlos, USA).

### Quantitative real-time polymerase chain reaction (RT-qPCR)

Total RNA from large intestines was extracted using TRI Reagent (Invitrogen, Carlsbad, CA, USA) and was quantified using a NanoDrop 2000 (Thermo Fisher Scientific). cDNA was synthesized with Novoscript® Plus All-in-one-1^st^ Strand cDNA Synthesis SuperMix (Novoprotein, Shanghai, China), according to the manufacturer’s instructions. This cDNA was subsequently used for RT-qPCR analysis using specific validated primers (Takara, Japan) and SYBR qPCR Supermix Plus (Novoprotein) in eight straight tubes in the StepOnePlus instrument (Thermo Fisher Scientific). StepOnePlus^TM^ software (Thermo Fisher Scientific) was used to analyze the standards and carry out the quantification. β-actin mRNA was used as the normalizing gene. The mRNA levels for each gene were expressed as 2-^ΔΔCt^, denoting fold change. Primer sequences were as follows: β-actin (forward) 5′-GCCTCACTGTCCACCTTCCA-3′, (reverse) 5′-AGCCATGCCAATGTTGTCTCTT-3′; interleukin-1β (IL-1β) (forward) 5′-TGCCACCTTTTGACAGTGATG-3′, (reverse) 5′-ATACTGCCTGCCTGAAGCTC-3′; tumor necrosis factor α (TNF-α) (forward) 5′-GACGTGGAACTGGCAGAAGAG-3′, (reverse) 5′-TTGGTGGTTTGTGAGTGTGAG-3′; and zonula occludens-1 (Zo-1) (forward) 5′-AGCGAATGTCTAAACCTGGG-3′, (reverse) 5′-TCCAACTTGAGCATACACAGG-3′.

### Statistical analysis

Statistical analyses were performed with the GraphPad Prism 6.0 software. Data were analyzed with two-way ANOVA to evaluate interactions among the groups, and Tukey’s test was used to detect differences between groups and the repeated measures. Student’s *t* test also was used for comparing differences of the groups. The results are expressed as the mean ± SEM. Statistical significance was set at *P* < 0.05.

## Results

### Oral Pg induced dopaminergic neuronal degeneration in the SNpc of mutant LRRK2 mice

**Fig. 1:**
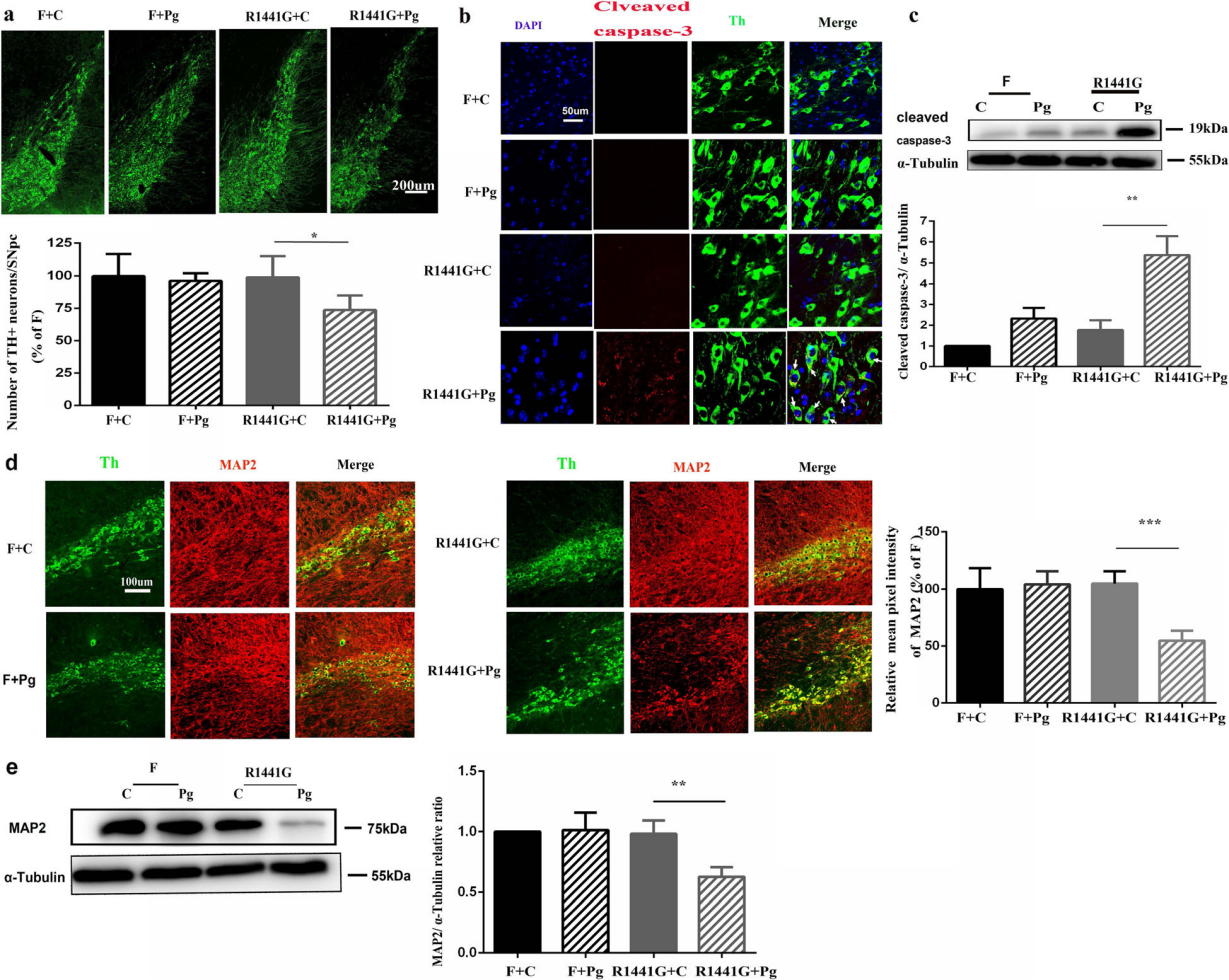
Oral Pg induced dopaminergic neuronal degeneration in the SNpc of mutant LRRK2 mice. **a** Representative images of immunofluorescence-stained coronal brain sections and quantification of TH number (a marker of dopaminergic neuron ) from the F + Pg and R1441G + Pg groups compared to F + C and R1441G + C mice. *n* = 4–5, *P < 0.05 (scale bar = 200 μm). Two-way ANOVA and Tukey’s test were used for analysis. **b** Representative images of TH+ dopaminergic neurons (green) and cleaved caspase-3 (red) (white arrows indicate co-localized cells) immunofluorescent staining from the SN in brain sections obtained from F + C, F + Pg, R1441G + Pg, and R1441G + C mice (scale bar = 50 μm). **c** Representative images of western blots of cleaved caspase-3 protein levels and quantitative analysis, which were done with the SN obtained from F + Pg and 1441 + Pg mice compared to F + C and 1441 + C mice. *n* = 4, **P < 0.01. Two-way ANOVA and Tukey’s test were used for analysis. **d** Representative images of immunofluorescence double staining with dendric marker MAP2 and comparison of dendric density from F + Pg, 1441 + Pg, F + C, and 1441 + C mice. *n* = 4–5, ***P < 0.001 (scale bar = 100 μm). Two-way ANOVA and Tukey’s test were used for analysis. **e** Representative images of western blots and quantitative analyses of MAP2 protein levels. Analyses of protein levels of MAP2 were done on the SN obtained from F + Pg and 1441 + Pg mice compared to F + C and 1441 + C mice. *n* = 4, **P < 0.01. Two-way ANOVA and Tukey’s test were used for analysis

To examine whether oral Pg can induce dopaminergic neuronal degeneration, Pg was administrated orally to FVBN mice and LRRK2 R1441G transgenic mice for a month. Immunofluorescence TH staining was used to examine loss of SNpc dopaminergic cells. We found that there was a significant loss of TH+ neurons in the SNpc in R1441G mice, but not in FVBN mice (Fig. 1a). Confocal immunofluorescence imaging revealed active caspase-3 in the cytoplasm and cell nucleus of TH+ SNpc dopaminergic neurons of LRRK2 R1441G mice, but not FVBN mice (Fig. 1b). Immunoblot further confirmed that protein level of cleaved active caspase-3 was greatly increased in the SN of LRRK2 R1441G mice after Pg treatment compared to FVBN mice (Fig. 1c). In addition, LRRK2 R1441G mice exhibited a significant reduction in the immunofluorescence intensity of SNpc MAP2+ dendrite (Fig. 1d), which was accompanied by the reduction in MAP2+ protein level (Fig. 1e). In contrast, the immunofluorescence intensity of SNpc MAP2+ dendrite was not significantly altered in FVBN + Pg. To further determine whether inflammation-induced neuronal loss is mutation specific, Pg was administered to mice overexpressing human wild-type LRRK2 (WT-OX). We found that there were no significant differences in TH+ number and expression levels of MAP2+ protein between Pg-treated WT-OX and WT-OX mice (Supplementary Figs. 1a, b), thereby suggesting that oral Pg-induced neurodegeneration was mutant LRRK2-dependent. Meanwhile, there were no significant differences in the rotarod and open field tests among these groups (Supplementary Fig. 1c).

### Oral Pg increased microglial activation in the SNpc of mutant LRRK2 mice

**Fig. 2:**
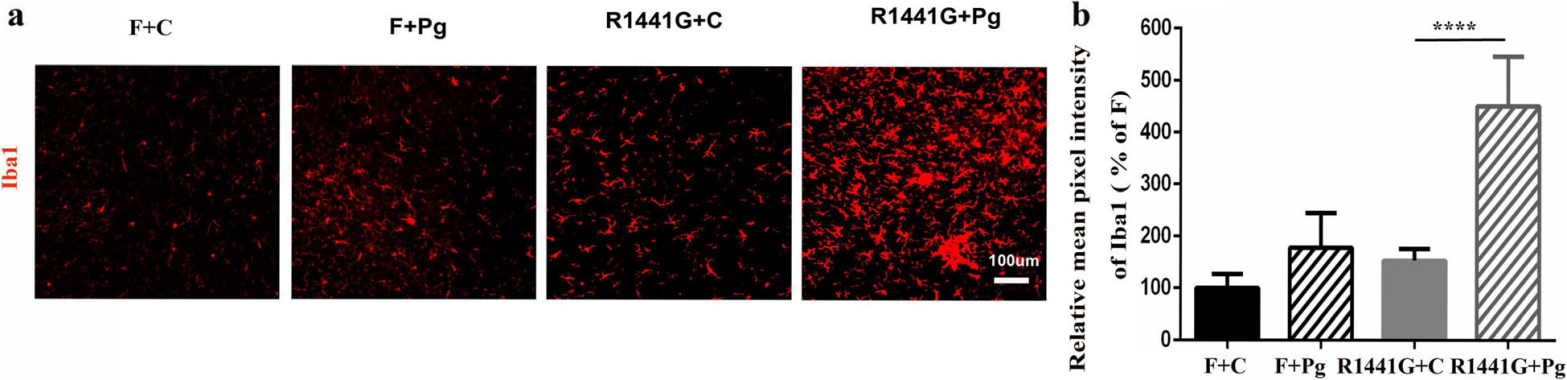
Oral Pg increased microglial activation in the SNpc of mutant LRRK2 mice. **a** Representative images of immunofluorescence staining with Iba1 (a marker of microglia) and comparison of Iba1 density (**b**) in the SN area from F + Pg and 1441 + Pg mice compared to F + C and 1441 + C mice. *n* = 4–5, ****P < 0.0001 (scale bar = 100 μm). Two-way ANOVA and Tukey’s test were used for analysis

Over-activation of microglia has been linked to neurodegeneration in PD [26, 27]. In the present study, there was a significant increase in the number of activated Iba1-positive microglia in the SNpc in R1441G mice compared to FVBN and WT-OX mice, 1 month following treatment with oral Pg (Fig. 2a, Supplementary Fig. 2a).

### Oral Pg increased LRRK2 activation in the SN of R1441G mice

**Fig. 3:**
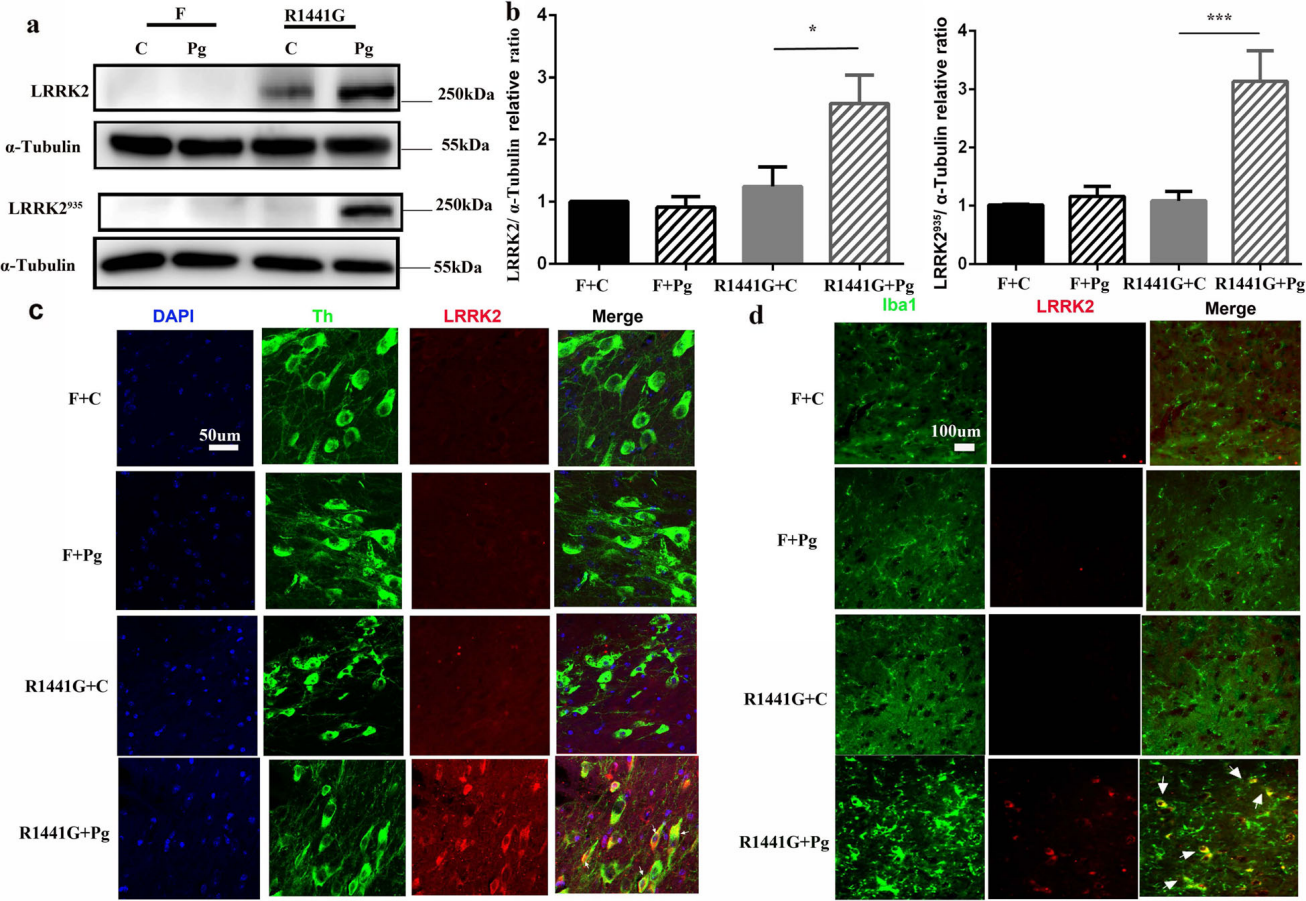
Oral Pg increased LRRK2 activation in the SN of R1441G mice. **a** Representative images of western blots of LRRK2 and LRRK^935^ protein levels and **b** quantitative analysis, which were done on the SN obtained from F + Pg and 1441 + Pg mice compared to F + C and 1441 + C mice. *n* = 4, ***P < 0.001,*P < 0.05. Two-way ANOVA and Tukey’s test were used for analysis. **c** Representative images of TH+ dopaminergic neuron by immunofluorescent double staining with LRRK2 (white arrows indicate co-localized cells) (scale bar = 50 μm). **d** Representative images of Iba1+ microglia by immunofluorescent double staining with LRRK2 (white arrows indicate the cells co-localized with LRRK2 and Iba1) (scale bar = 100 μm)

Mutant LRRK2 has been implicated in neuronal cell death and microglial inflammatory response of SNpc [5, 10]. In this study, both LRRK2 and LRRK2^p935^ were significantly increased in the SN of Pg-treated R1441G mice compared to Pg-treated FVBN mice (Fig. 3a, b). Although LRRK2 protein expression was also increased in the SN of Pg-treated WT-OX mice, LRRK2^p935^ was not altered in WT-OX after Pg treatment (Supplementary Fig. 2c). Double immunofluorescence staining using anti-LRKK2, anti-TH, and anti-Iba1 was performed to visualize the co-localization of LRRK2 in SNpc dopaminergic neurons and microglia. Consistent with western blots, the immunosignal of LRRK2 was evident in Pg-treated R1441G mice. In addition, LRRK2 was partially co-localized with TH+ neurons and Iba1+ microglia (Fig. 3c, d).

### Mutant LRRK2 exacerbated Pg-induced peripheral IL-17A secretion and IL-17RA upregulation

**Fig. 4:**
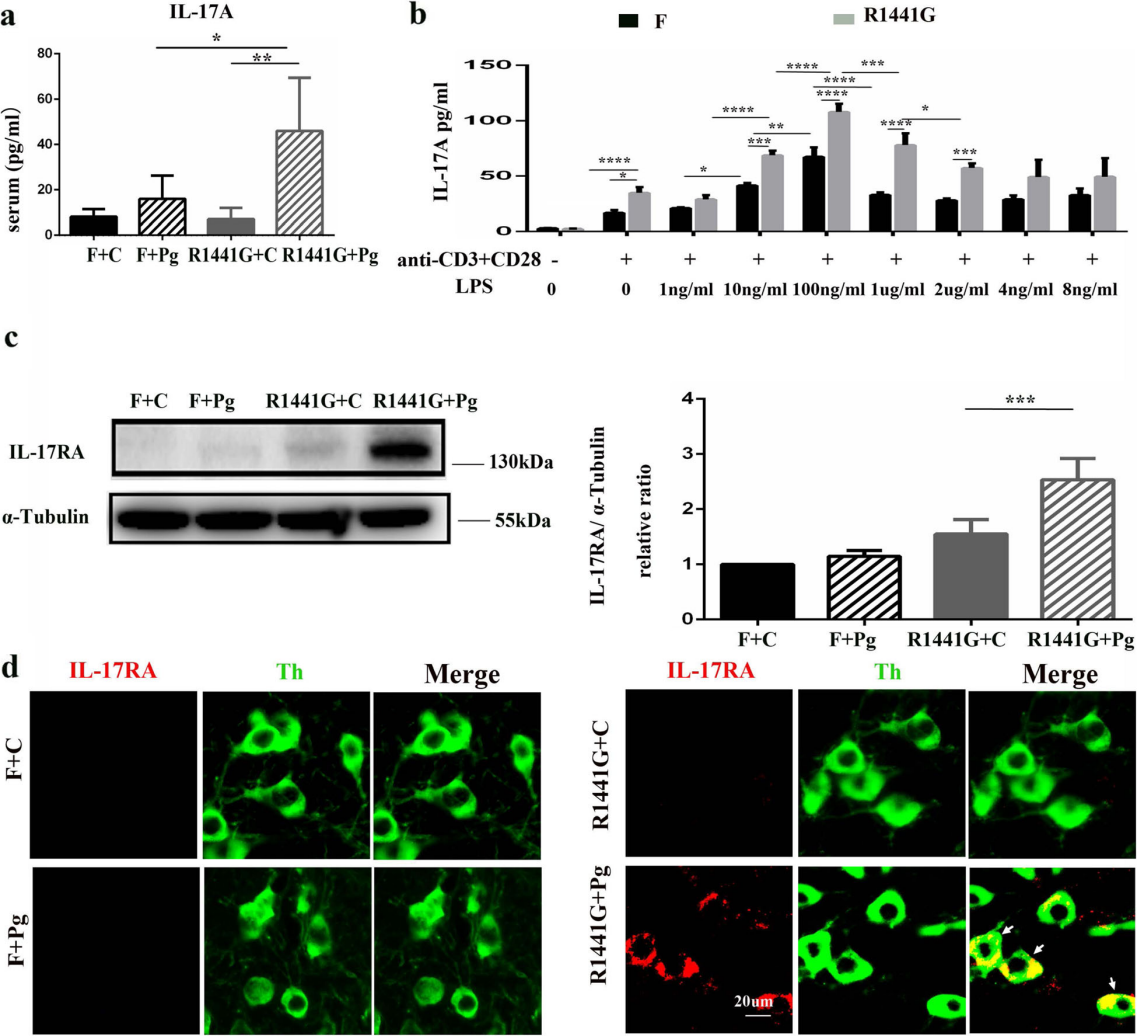
Mutant LRRK2 exacerbated Pg-induced peripheral IL-17A secretion and IL-17RA upregulation. The mice were equally divided into four groups, F + C, F + Pg, 1441 + C, and 1441 + Pg. **a** IL-17A protein levels in serum were examined using multiplex cytokine and chemokine analysis. *n* = 4–5, *P < 0.05. Two-way ANOVA and Tukey’s test were used for analysis. **b** Pg-treated mononuclear cells were stimulated for about 24 h and the IL-17A protein levels in the supernatant were measured by ELISA. *n* = 4, *P < 0.05, **P < 0.01, ***P < 0.001, ****P < 0.0001. Two-way ANOVA and Tukey’s test were used for analysis. **c** Representative images of western blot of IL-17RA obtained from SN tissue and quantitative analysis. *n* = 4, ***P < 0.001. Two-way ANOVA and Tukey’s test were used for analysis. **d** Representative images of co-localization of TH (green) and IL-17RA (red) from four groups, F + C, F + Pg, 1441 + C, and 1441 + Pg (scale bar = 20 μm)

Several cytokines such as INF-γ, IL-lα, IL-4, IL-13, TGF-β, and IL-17A have been reported to increase LRRK2 expression. Among them, INF-γ, IL-lα, and IL-17A could mediate dopaminergic neurodegeneration [28–31]. Therefore, we examined the serum levels of IL-17A, INF-γ, and IL-lα in animals receiving either CMC or Pg. There was no significant difference in IL-17A between FVBN and R1441G mice following CMC treatment (Fig. 4a). Furthermore, there were no significant differences in serum INF-γ and IL-lα between R1441G and FVBN mice following Pg treatment (Supplementary Fig. 3a, b). However, serum IL-17A was significantly increased in R1441G mice compared to FVBN mice following oral administration of Pg (Fig. 4a). Consistently, IL-17A was significantly increased in the supernatant of splenic mononuclear cells from R1441G but not FVBN mice after anti-CD3+CD28 stimulation (Fig. 4b). In addition, IL-17A was significantly higher in R1441G cells than in FVBN cells after anti-CD3+CD28+ LPS stimulation (Fig. 4b). LPS induced IL-17A in a bell-shaped manner, and the peak response was observed at 100 ng/ml. Given that high concentrations of LPS have been shown to induce cell death, LPS-induced toxicity may account for the lack of response to high doses of LPS in splenic cells [32, 33] (Fig. 4b). In the present study, T cells and CCR2+ monocytes were not detected in the brain. In addition, IL-17A protein level remained unchanged (Supplementary Fig. 2b, Supplementary Fig. 3c, e). Thus, it is less likely that peripheral T cells and monocytes directly mediated dopaminergic neuron loss. Instead, IL-17RA protein level was elevated in the SN of R1441G mice with Pg compared with FVBN mice (Fig. 4c). IL-17RA was co-localized with TH in the SN of R1441G mice with Pg. However, IL-17RA was not co-localized with Iba-1 (Fig. 4d, Supplementary Fig. 3d).

### Pg treatment increased the accumulation of α-synuclein in neurons of the colon and induced activation of LRRK2

**Fig. 5:**
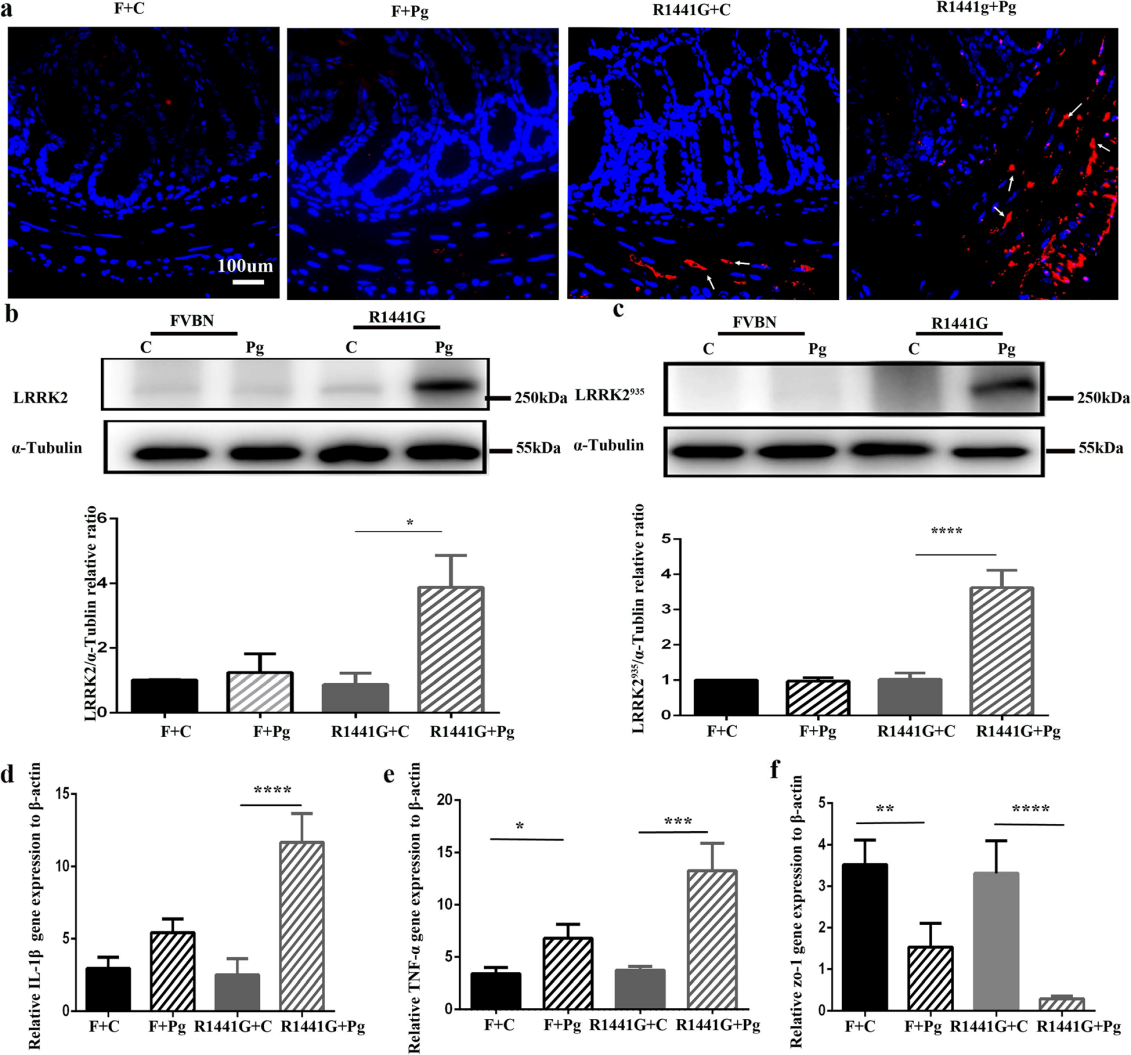
Pg treatment increased the accumulation of α-synuclein in neurons of the colon and caused activation of LRRK2. **a** Representative images of α-synuclein (red) (white arrows) immunofluorescent staining from the colon obtained from F + C, F + Pg, 1441 + Pg, and 1441 + C mice (scale bar = 100 μm). Representative images of western blots of **b** LRRK2 and **c** LRRK^935^ protein levels, and quantitative analysis, which were done with the colon tissue obtained from the F + Pg and 1441 + Pg groups and compared with F + C and 1441 + C mice. *n* = 4, ****P < 0.0001,*P < 0.05. Two-way ANOVA and Tukey’s test were used for analysis. Comparison of relative **d** IL-1β, **e** TNF-α, and **f** Zo-1 gene expression levels in the colon from F + C, F + Pg, 1441 + Pg, and 1441 + C mice. *n* = 6–8, *P < 0.05, **P < 0.01, ***P < 0.001, ****P < 0.0001. Two-way ANOVA and Tukey’s test were used for analysis

Emerging evidence suggests that α-synuclein accumulates in neurons of the gut prior to the brain in PD [17, 34]. Although histological analysis revealed normal morphology of the colon and small intestine in all groups (Supplementary Fig. 4a), we found that the α-synuclein in the myenteric plexus of the colon was higher in Pg-treated R1441G mice than in control mice (Fig. 5a). There was no detectable α-synuclein in the brain and small intestine (data not shown). Furthermore, immunoblot analysis demonstrated a significant increase in LRRK2 and LRRK2^935^ protein levels in the colon of Pg-treated R1441G mice compared to each of the other three groups of mice. (Fig. 5b, c) Besides, oral administration of Pg led to a significant increase in mRNA expression of TNF-a and IL-1β along with the significant decrease of Zo-1 in the colon of R1441G mice, but not FVBN mice (Fig. 5d–f).

## Discussion

LRRK2 is highly expressed in immune cells, and mutation of LRRK2 has been linked to both intestinal inflammatory disease and PD [5, 35, 36]. In this study, we investigated the contribution of oral Pg to the pathogenesis of mutant LRRK2-associated PD in LRRK2 (R1441G) mice. Although oral Pg induced a mild inflammatory response in the intestine, it caused a significant loss of dopaminergic neurons and profound microglial activation in the SNpc. In addition, oral Pg resulted in an IL-17A immune response in the peripheral system and upregulation of IL-17RA protein levels of dopaminergic neurons, thereby suggesting that oral Pg may mediate a correlation between IL-17A and DA neurodegeneration LRRK2-associated PD. Furthermore, these oral Pg-mediated harmful effects were accompanied by an increase in LRRK2^935^ expression, an indirect marker of LRRK2 kinase activity, which suggests the involvement of LRRK2 kinase in Pg-induced neuropathogenesis in LRRK2-associated PD.

Systemic inflammation has been shown to induce dopaminergic neuronal death through activation of LRRK2 [6]. We consistently found that dopaminergic degeneration was evident in R1441G animals following Pg administration. This event might be mediated by aberrant LRRK2 kinase, as evidenced by an increase in LRRK2^935^ expression in both the brain and colon. Interestingly, Pg-induced expression of LRRK2^935^ was associated with profound activation of microglia, whereas the gut morphology was much less affected in R1441G mice. Thus, it is intriguing how oral Pg induces neuroinflammation in the brain.

In PD, α-synuclein is considered to play a pivotal role in brain-gut-microbiota axis interactions [34, 37]. Gut inflammation induces expression of α-synuclein, and the latter travels along with the vagus nerve to initiate the process of α-synuclein misfolding in the brain, which leads to neuroinflammation [38]. Previously, LRRK2 activity has been shown to enhance expression of α-synuclein [39, 40]. Pg-mediated LRRK2 activation consistently induced expression of α-synuclein in the colon of R1441G mice. However, α-synuclein levels in the brain were not significantly different between FVBN and R1441G mice, thereby suggesting that α-synuclein may not be responsible for activation of microglia. This is in contrast to a recent observation that injection of α-synuclein fibrils in the gut mediates the spread of pathologic α-synuclein in the brain via the vagus nerve [41]. Use of different animal models may explain the discrepancy between our study and the previous study. Indeed, α-synuclein fibrils can effectively seed the formation of Lewy body-like inclusions due to their high aggregation propensity.

Alternatively, gut-mediated systemic inflammation may induce brain inflammation via circulating cytokines [38, 42]. Interestingly, it has been reported that circulating cytokines including INF-γ, IL-lα, and IL-17A not only increase LRRK2 expression but also mediate dopaminergic neurodegeneration. Pg is an oral pathogen that is known to be able to induce a systemic inflammatory response [43, 44]. Oral bacteria in the gut may also lead to systemic diseases through gut inflammation [14, 15]. Indeed, Nakajima et al. have demonstrated that oral Pg may induce systemic inflammation associated with alteration of gut inflammation [14]. Inflammation in the gut can disrupt gut barrier which allows toxic molecules such as LPS into the systemic circulation, thereby triggering systemic inflammation [16, 42]. Consistently, we found that oral Pg induced IL-1β and TNF-a in the colon and reduced epithelial barrier protein Zo-1. In addition, serum IL-17A but not INF-γ nor IL-lα was significantly increased in R1441G mice animals with oral Pg, indicating that serum IL-17A might have an important role in the pathogenesis of LRRK2-PD. In the present study, Pg-induced IL-17A is less likely derived from peripheral intestinal related lymphoid tissues, such as payer’s patches and mesenteric lymph node, because IL-17A was not detected in the colon in the present study (data no shown). Recently, Kozina et al. have shown that LPS treatment can induce peripheral and central immune responses leading to neurodegeneration in LRRK2-associated PD [6]. Pg-derived LPS, the main pathogenic factor of Pg, has been shown to mediate periodontitis-associated systemic inflammation [45]. In the present study, Pg-derived LPS could increase IL-17A levels in splenic mononuclear cells from R1441G mice. Given that IL-17A is primarily secreted by a distinct CD4+ T cell subset, known as T helper 17 cells (Th17), the present data suggests that Pg-LPS may promote splenic Th17 differentiation. In addition to Th17 cells, other types of cells such as γδ T, CD8+ T cells, B cells, and NKT can also produce IL-17A. Further studies should be conducted to determine the cell types producing IL-17A [46–48].

Elevated IL-17A level has been reported in the serum from patients with PD [20, 21]. Emerging evidence indicates that IL-17A can induce neuroinflammation in animal models and PD patients [49–52]. Peripheral IL-17A has been reported to disrupt and cross the blood-brain barrier [52]. However, neither IL-17A nor Th17 cells could be detected in the brain in our study. IL-17RA is required for the biological activity of IL-17A [53]. In our study, we found that IL-17RA was increased in the dopaminergic neurons of the SN. Several studies have shown that IL-17 could trigger neuronal death through IL-17RA [21]. Thus, it is likely that serum IL-17A may mediate neuronal death through interaction with IL-17RA in Pg-induced R1441G mice. Interestingly, IL-17A has been reported to mediate dopaminergic neuron degeneration via IL-17RA in microglia in a previous study [51]. However, we did not detect any expression of IL-17RA in microglia, although reactive microglia were evident in Pg-induced R1441G mice (Supplementary Fig. 2g). The differing results may be due to the use of different models in the experiments. However, it should be noted that the present data only suggests a correlation between serum IL-17A and neurodegeneration. Future studies are needed to determine whether blocking IL-17A can attenuate Pg-induced neurodegeneration in LRRK2-associated PD. In addition, LPS treatment alone has been shown to induce peripheral and central immune responses leading to neurodegenerative in LRRK2-associated PD [6]. Thus, we could not rule out some other connections that are responsible for neurodegeneration. We believe that oral Pg may induce complex immune responses involving a variety of signal pathways in LRRK2-associated PD. Therefore, future studies with comprehensive profiling of circulating cytokines are necessary to identify the neurodegenerative pathways induced by oral Pg.

Microglia in a state of heightened reactivity have a vital role in PD [54]. Evidence suggests that mutant LRRK2 may enhance microglial process outgrowth and inflammatory response leading to chronic damage of dopaminergic neurons [55, 56]. Our study consistently showed a significant increase in Iba1 number in R1441G LRRK2 mice. Besides, our study showed LRRK2 co-localization in microglia and neurons. However, the precise role of LRRK2 in Pg-induced neurodegeneration should be examined in further studies using animal bearing a LRRK2 variant that cannot be phosphorylated.

## Conclusions

Our results indicate that oral Pg impairs gut permeability and induces an increase of peripheral IL-17A in the R1441G mice, which might be associated with neuronal death and neuroinflammation. In conclusion, the present study suggests a potential role of oral Pg-induced inflammation in the pathophysiology of LRRK2-associated PD.

## Supplementary Information

**Additional file 1:** **Figure S1.** (a) Representative images of immunofluorescence-stained coronal brain sections and quantification of TH number (a marker of dopaminergic neuron ) from WT-OX + Pg compared to WT-OX + C mice. n = 4–5. (Scale bar = 200 μm.) A Student’ t test was used for analysis. (b) Representative images of immunofluorescence double staining with dendric marker MAP2 and comparison of dendric density from WT-OX + Pg compared to WT-OX + C mice. n = 4–5. (Scale bar =100 μm.) A Student’ t test was used for analysis. (c) Latency to fall in the rotarod test (left panel) and locomotor activity distance moved in the open field test (right panel) from F + C, F + Pg, 1441 + Pg, and 1441 + C mice, n = 8. Two-way ANOVA and Tukey’s test were used for analysis.

**Additional file 2: Figure S2.** (a) Representative images of immunofluorescence staining with Iba1 (a marker of microglia) and comparison of Iba1 density in the SN area from WT-OX + Pg compared to WT-OX + C mice. n = 4-5. (Scale bar = 100 μm). A Student’ t test was used for analysis. (b) Representative images of co-localization of CCR2 (red) and Th (green) in the SN from Pg-treated R1441G mice. (Scale bar = 100 μm). (c) Representative images of western blots of LRRK2 (left panel) and LRRK^935^ protein levels (right panel), which were done with the SN obtained from WT-OX + Pg and WT-OX + C mice.

**Additional file 3: Figure S3.** INF-γ and IL-1α protein levels in serum (a, b) were examined using multiplex cytokines and chemokines analysis from F + C, F + Pg, 1441 + C, and 1441 + Pg, n = 4–5. Two-way ANOVA and Tukey’s test were used for analysis. (c) Representative images of western blots of IL-17A obtained from SN tissue and quantitative analysis, n = 4. Two-way ANOVA and Tukey’s test were used for analysis. (d) Representative images of co-localization of Iba1 (red) and IL-17RA (green) in the SN from Pg-treated R1441G mice. (Scale bar = 20 μm). (e) Representative images of co-localization of CD3 (red) and Th (green) in the SN from Pg-treated R1441G mice. (Scale bar = 100 μm).

**Additional file 4:** **Figure S4.** (a) Representative images of histopathology of the colon and small intestine obtained from F + C, F + Pg, 1441 + Pg, and 1441 + C mice. (Scale bar = 100 μm.)

## Data Availability

All data generated or analyzed during this study are included in this published article [and its supplementary information files].

## Abbreviations

LRRK2: Leucine-rich repeat kinase 2
Pg: *Porphyromonas gingivalis*
LPS: Lipopolysaccharide
IBA-1: Allograft inflammatory factor 1
IL-17A: Interleukin-17A
IL-1β: Interleukin-1β
Zo-1: Zonula occludens-1
PD: Parkinson’s disease
SNpc: Substantia nigra pars compacta
TH: Tyrosine hydroxylase
TNF-α: Tumor necrosis factor α
CMC: Carboxymethyl cellulose
WT-OX: Overexpressing human wild-type
PCR: Polymerase chain reaction
PBS: Phosphate-buffered saline
CMC: Carboxymethyl cellulose
