# Supplementary figures and images for: Oral P. gingivalis impairs gut permeability and mediates immune responses associated with neurodegeneration in LRRK2 R1441G mice
Source: J Neuroinflammation. 2020 Nov 19;17:347. doi: 10.1186/s12974-020-02027-5 (PMC7677837; doi:10.1186/s12974-020-02027-5)

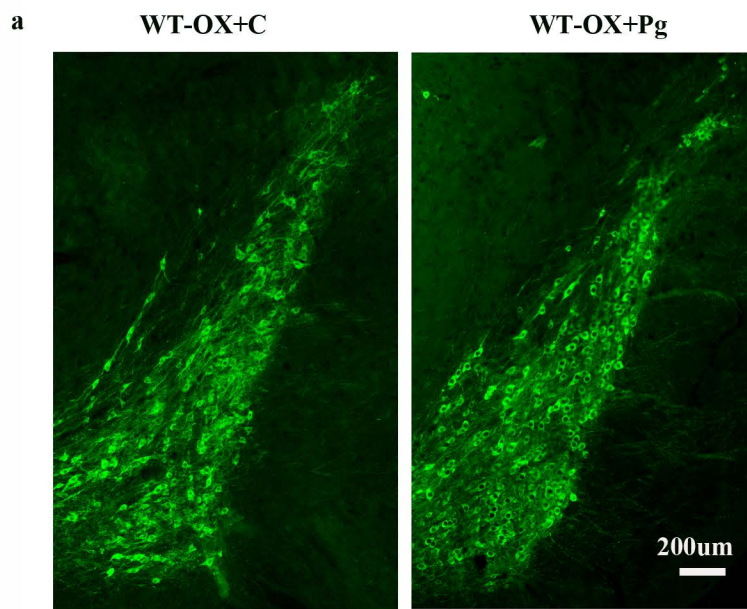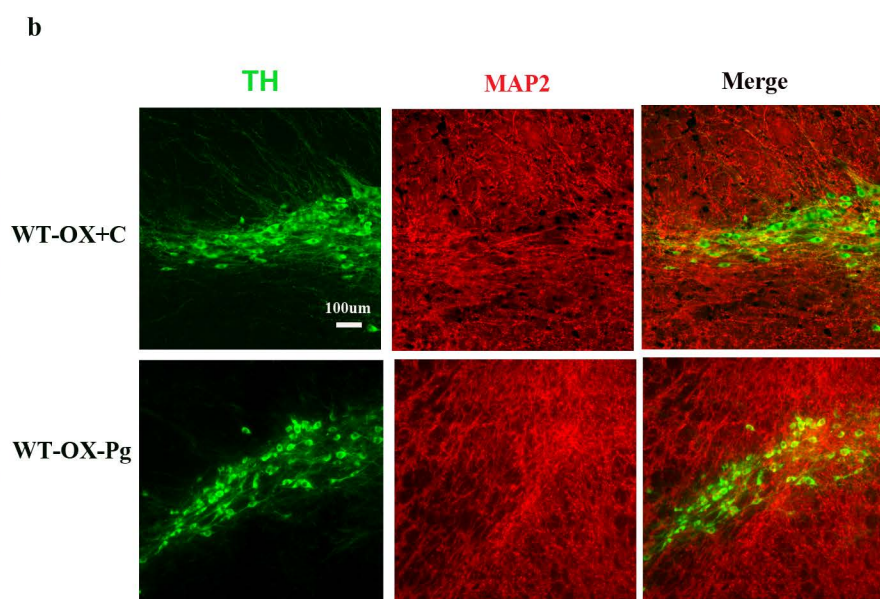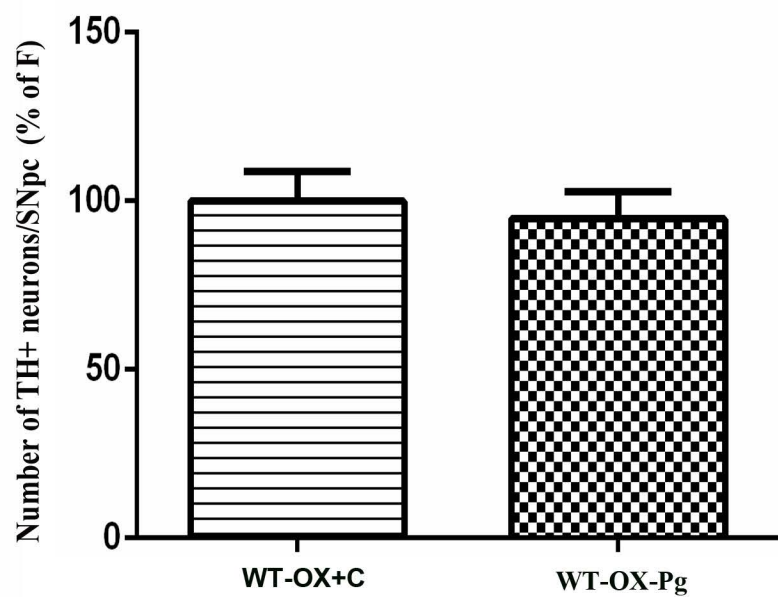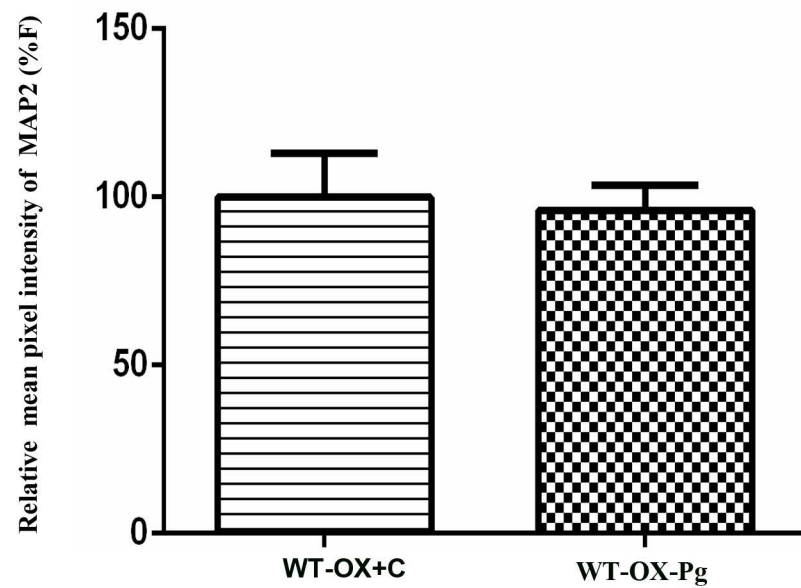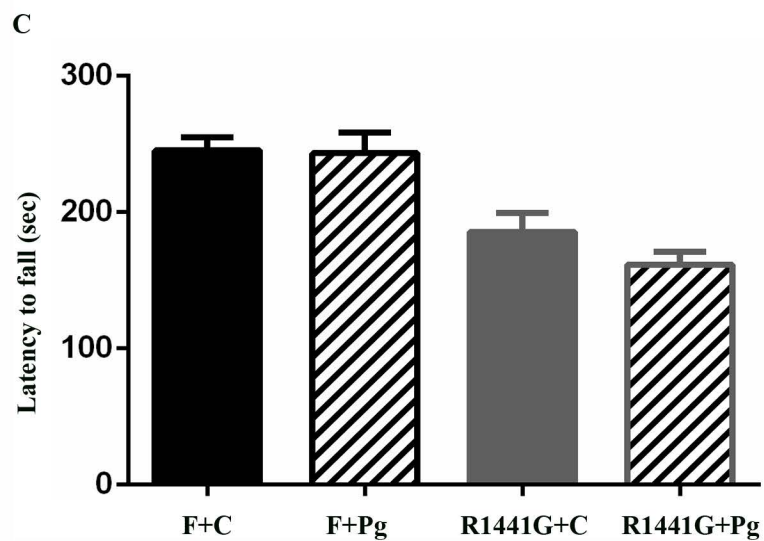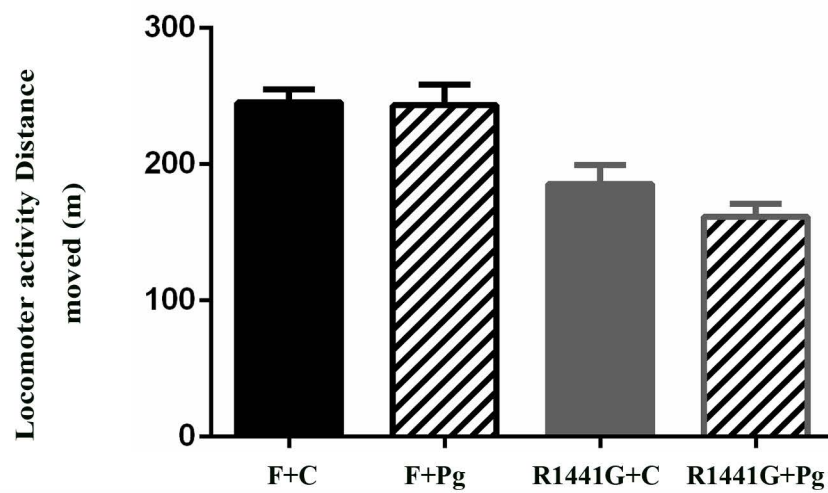

Supplement: Supplementary file 1 — Additional file 1: Figure S1. (a) Representative images of immunofluorescence-stained coronal brain sections and quantification of TH number (a marker of dopaminergic neuron ) from WT-OX + Pg compared to WT-OX + C mice. n = 4–5. (Scale bar = 200 μm.) A Student’ t test was used for analysis. (b) Representative images of immunofluorescence double staining with dendric marker MAP2 and comparison of dendric density from WT-OX + Pg compared to WT-OX + C mice. n = 4–5. (Scale bar =100 μm.) A Student’ t test was used for analysis. (c) Latency to fall in the rotarod test (left panel) and locomotor activity distance moved in the open field test (right panel) from F + C, F + Pg, 1441 + Pg, and 1441 + C mice, n = 8. Two-way ANOVA and Tukey’s test were used for analysis. [file 12974_2020_2027_MOESM1_ESM.pdf]

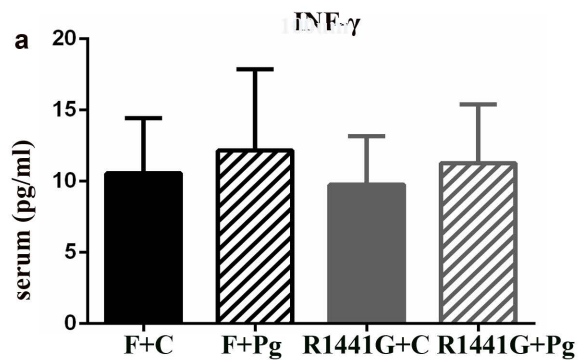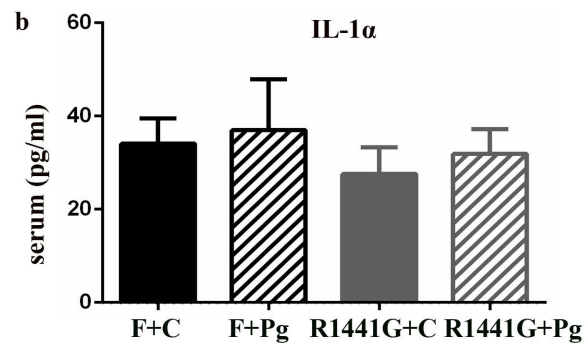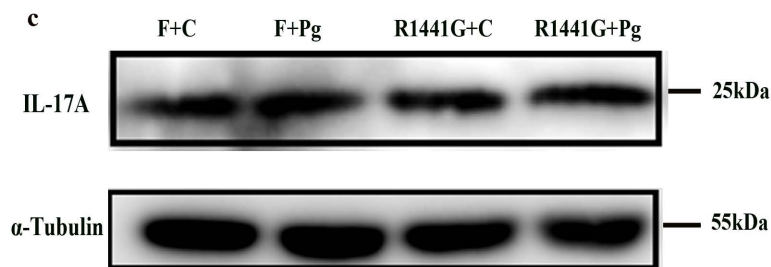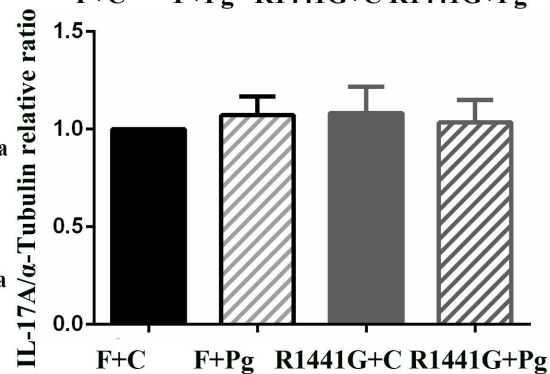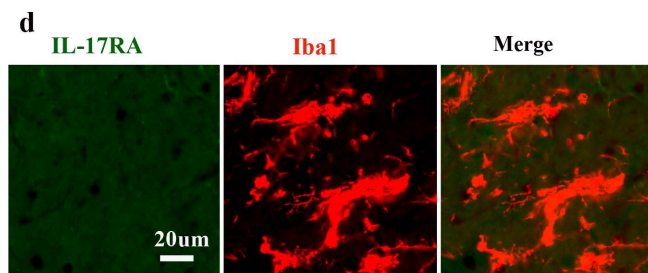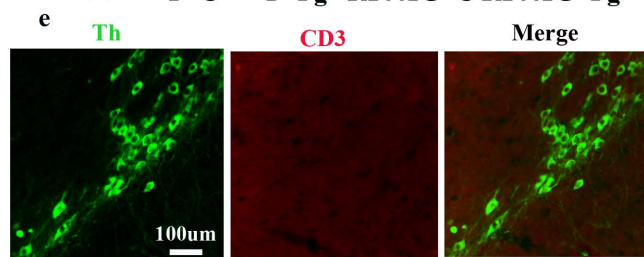

Supplement: Supplementary file 3 — Additional file 3: Figure S3. INF-γ and IL-1α protein levels in serum (a, b) were examined using multiplex cytokines and chemokines analysis from F + C, F + Pg, 1441 + C, and 1441 + Pg, n = 4–5. Two-way ANOVA and Tukey’s test were used for analysis. (c) Representative images of western blots of IL-17A obtained from SN tissue and quantitative analysis, n = 4. Two-way ANOVA and Tukey’s test were used for analysis. (d) Representative images of co-localization of Iba1 (red) and IL-17RA (green) in the SN from Pg-treated R1441G mice. (Scale bar = 20 μm). (e) Representative images of co-localization of CD3 (red) and Th (green) in the SN from Pg-treated R1441G mice. (Scale bar = 100 μm). [file 12974_2020_2027_MOESM3_ESM.pdf]

FVBN

a

intestinal

colon

c

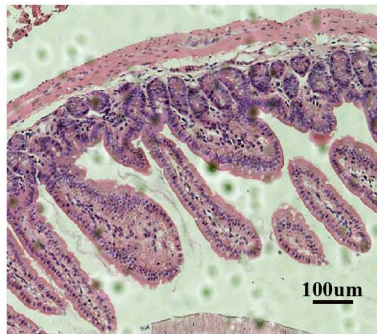

Pg

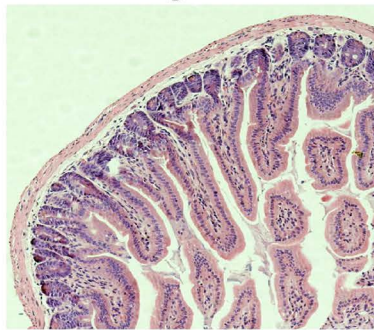

R1441G

intestinal

colon

c

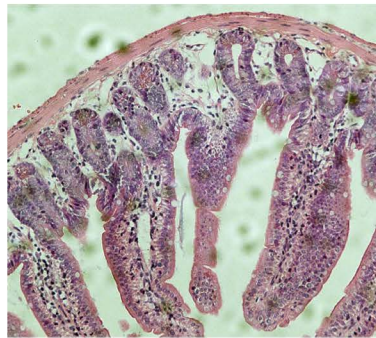

Pg

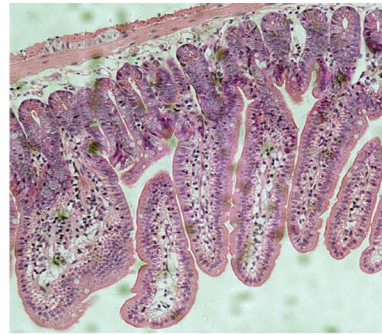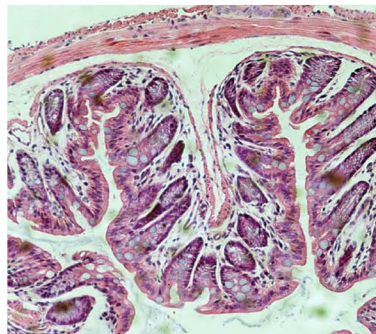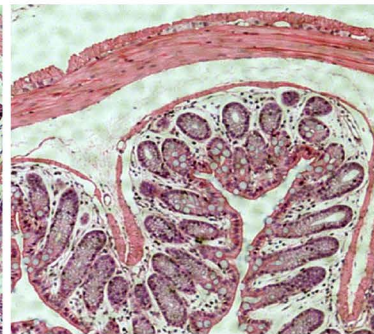

Supplement: Supplementary file 4 — Additional file 4: Figure S4. (a) Representative images of histopathology of the colon and small intestine obtained from F + C, F + Pg, 1441 + Pg, and 1441 + C mice. (Scale bar = 100 μm.) [file 12974_2020_2027_MOESM4_ESM.pdf]
