# Supplementary material for: Oral P. gingivalis impairs gut permeability and mediates immune responses associated with neurodegeneration in LRRK2 R1441G mice
Source: J Neuroinflammation. 2020 Nov 19;17:347. doi: 10.1186/s12974-020-02027-5 (PMC7677837; doi:10.1186/s12974-020-02027-5)
Supplement: Supplementary file 2 — Additional file 2: Figure S2. (a) Representative images of immunofluorescence staining with Iba1 (a marker of microglia) and comparison of Iba1 density in the SN area from WT-OX + Pg compared to WT-OX + C mice. n = 4-5. (Scale bar = 100 μm). A Student’ t test was used for analysis. (b) Representative images of co-localization of CCR2 (red) and Th (green) in the SN from Pg-treated R1441G mice. (Scale bar = 100 μm). (c) Representative images of western blots of LRRK2 (left panel) and LRRK935 protein levels (right panel), which were done with the SN obtained from WT-OX + Pg and WT-OX + C mice. [file 12974_2020_2027_MOESM2_ESM.pdf]

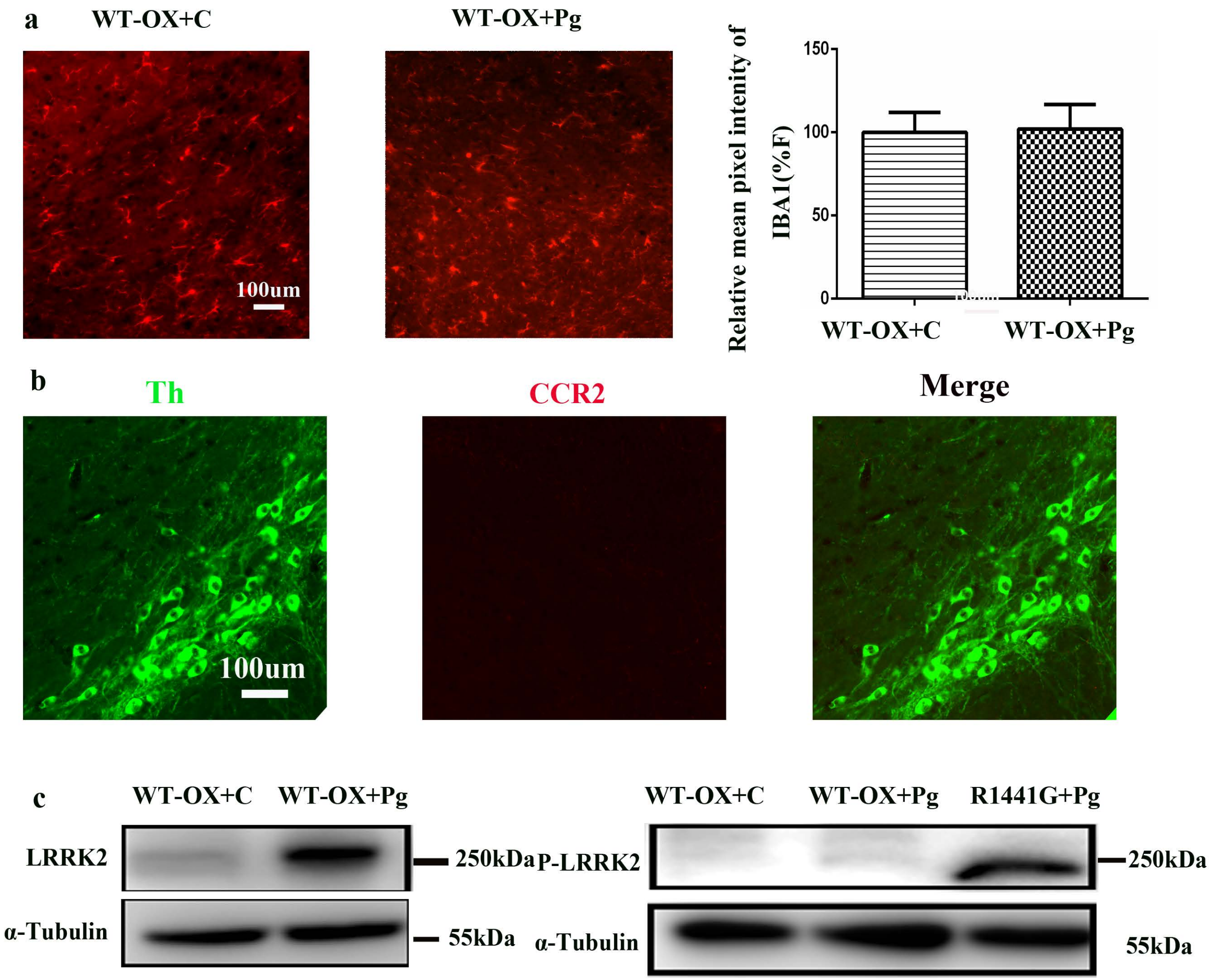

a
WT-OX+C
WT-OX+Pg
100um
Relative mean pixel intenity of IBA1(%F)
150
100
50
0
WT-OX+C
WT-OX+Pg
b
Th
CCR2
Merge
100um
c
WT-OX+C
WT-OX+Pg
LRRK2
250kDa
α-Tubulin
55kDa
WT-OX+C
WT-OX+Pg
R1441G+Pg
P-LRRK2
250kDa
α-Tubulin
55kDa
